# Impact of a Behavioral Lifestyle Intervention on Dietary Habits, Anthropometric and Psychological Outcomes, and Biomarkers of Oxidative Stress and Telomere Length in Adults

**DOI:** 10.3390/nu18152501

**Published:** 2026-08-03

**Authors:** Panagiota Galatoula, Panagiotis Pipelias, Vasileios Mantikas, Argyro Papadopoulou, George Chrousos, Flora Bacopoulou, Michail Mykoniatis, Christina Darviri

**Affiliations:** 1 Postgraduate Program “The Science of Stress and Health Promotion”, School of Medicine, National and Kapodistrian University of Athens, 10679 Athens, Greece; pgalatoula@med.uoa.gr (P.G.); argyro.papadopoulou@yahoo.com (A.P.); chrousge@med.uoa.gr (G.C.); mikemikon@med.uoa.gr (M.M.); cdarviri@med.uoa.gr (C.D.); 2 Department of Geriatrics, Gesundheitszentrum, 8157 Dielsdorf, Switzerland; mantikasvasileios@gmail.com; 3 University Research Institute of Maternal and Child Health and Precision Medicine, School of Medicine, National and Kapodistrian University of Athens, 11527 Athens, Greece; 4 Adolescent Health Care, National and Kapodistrian University of Athens, Aghia Sophia Children’s Hospital, 11527 Athens, Greece; fbacopoulou@med.uoa.gr; 5 Clinical and Translational Research Endocrine Unit, School of Medicine, National and Kapodistrian University of Athens, 11528 Athens, Greece; 6 Clinic for Assessment of Learning Difficulties, Center for Adolescent Medicine, First Department of Pediatrics, School of Medicine, National and Kapodistrian University of Athens, Aghia Sophia Children’s Hospital, 11527 Athens, Greece

**Keywords:** Pythagorean Self-Awareness Intervention, lifestyle intervention, oxidative stress, telomere length, psychological health, cognitive function, body composition

## Abstract

**Background/Objectives**: Lifestyle-related behaviors and chronic stress are key determinants of metabolic health, psychological well-being, and biological aging. Structured behavioral interventions that enhance self-awareness and self-regulation may promote sustained improvements across multiple health domains. This study aimed to evaluate the effects of a six-week Pythagorean Self-Awareness Intervention (PSAI) on dietary habits, anthropometric and psychological outcomes, and biomarkers of oxidative stress and telomere length in adults. **Methods**: This quasi-experimental pre–post intervention study included 30 apparently healthy adults. Participants underwent baseline assessments followed by a six-week Pythagorean Self-Awareness Intervention (PSAI) and were reassessed post-intervention. Outcomes included Mediterranean diet adherence (MedDiet Score), anthropometric and body composition measures, validated psychological questionnaires (PSS-14, DASS-21, RSES, HLPCQ, HLC, STAXI, SSGS, PSQI), cognitive performance (SDMT, CVLT-II, BVMT-R), serum oxidative stress and antioxidant capacity, and leukocyte telomere length (T/S ratio). Statistical analyses were performed using paired-samples Student *t*-tests or Wilcoxon signed-rank tests, with significance set at *p* < 0.05. **Results**: Significant improvements were observed in Mediterranean diet adherence (*p* = 0.001), along with reductions in body weight, BMI, waist and hip circumference, and intermuscular adipose tissue (all *p* < 0.05). Psychological outcomes improved significantly, including reduced perceived stress (PSS-14, *p* = 0.004) and depressive symptoms (DASS-21, *p* = 0.005), and improved self-esteem (RSES, *p* = 0.003) and sleep quality (PSQI, *p* < 0.001). Cognitive performance improved across all domains, including information processing speed, verbal learning, and visuospatial memory (all *p* < 0.05). Regarding biological markers, oxidative stress decreased (*p* = 0.023), and antioxidant capacity (*p* = 0.009), and leukocyte telomere length increased (*p* < 0.001), indicating improved redox balance and cellular aging profiles. **Conclusions**: The six-week Pythagorean Self-Awareness Intervention (PSAI) was associated with significant improvements in dietary habits, anthropometric and psychological outcomes, cognitive performance, and biomarkers of oxidative stress and telomere length. These findings suggest that participation in a structured self-awareness-based intervention was associated with multidomain improvements in this cohort. However, given the quasi-experimental design without a control group, these findings should be considered preliminary and require confirmation in adequately powered randomized controlled trials.

## 1. Introduction

Lifestyle-related chronic diseases, including obesity, metabolic dysfunction, cardiovascular disease, and type 2 diabetes mellitus, represent a major global public health challenge [1,2]. Their development and progression are strongly influenced by modifiable lifestyle factors, including dietary habits [3], physical inactivity [4], inadequate sleep [5], and chronic psychological stress [6]. These behavioral determinants interact through interconnected physiological and psychological pathways, ultimately shaping metabolic health, quality of life, and biological aging processes [7].

Among these factors, chronic psychological stress plays a central role in the pathophysiology of metabolic dysfunction [8]. Persistent stress exposure leads to dysregulation of key stress-response systems, particularly the hypothalamic–pituitary–adrenal (HPA) axis and the autonomic nervous system [9]. Activation of the HPA axis induces cortisol secretion, a glucocorticoid hormone essential for maintaining homeostasis under acute stress conditions [10,11]. Although acute cortisol responses are adaptive and necessary for survival [12], chronic activation of the HPA axis may result in sustained cortisol dysregulation and contribute to disturbances in metabolic, endocrine, immune, and gastrointestinal function [13,14,15].

Accumulating evidence indicates that chronic stress and cortisol dysregulation are associated with visceral adiposity, impaired sleep quality, systemic inflammation, insulin resistance, mood disturbances, and broader metabolic dysfunction [16,17,18]. Furthermore, chronic stress has been linked to increased allostatic load, reflecting the cumulative physiological burden imposed by repeated or prolonged exposure to stressors [19]. Through combined neuroendocrine, inflammatory, and behavioral pathways, chronic stress may accelerate biological aging and increase vulnerability to chronic disease [20].

Importantly, stress-related physiological dysregulation is closely intertwined with behavioral adaptations [21]. Under conditions of chronic stress, individuals frequently exhibit emotional eating, poor dietary choices, reduced physical activity, sleep disruption, and lower adherence to healthy lifestyle behaviors [22]. These maladaptive behaviors further exacerbate metabolic dysfunction, adipose tissue dysregulation, and systemic inflammation, thereby reinforcing a bidirectional cycle between stress and poor health outcomes [23,24,25].

Nutritional behaviors play a central role in maintaining metabolic homeostasis and overall health [26]. In recent years, considerable attention has focused on the Mediterranean dietary pattern, which is characterized by high consumption of fruits, vegetables, legumes, whole grains, olive oil, and moderate intake of fish and dairy products [27,28,29]. Adherence to the Mediterranean diet has consistently been associated with favorable cardiometabolic outcomes, lower inflammatory burden, improved body composition, and enhanced psychological well-being [30,31]. However, despite widespread knowledge regarding the benefits of healthy eating patterns, achieving and maintaining long-term dietary adherence remains challenging [32]. Sustainable behavior change depends not only on nutritional knowledge but also on psychological processes such as self-awareness, emotional regulation, motivation, and self-regulation [33].

Beyond diet, increasing evidence highlights the importance of broader lifestyle behaviors, including sleep, physical activity, and meal timing, in the regulation of metabolic health [34,35]. Sleep disruption and circadian misalignment have been associated with obesity, impaired glucose metabolism, inflammation, altered cortisol secretion, and reduced psychological well-being [36,37]. Similarly, sedentary behavior has been linked to adipose tissue dysfunction, insulin resistance, and chronic low-grade inflammation [38], whereas regular physical activity appears to exert protective effects through improvements in metabolic regulation, body composition, and inflammatory balance [39,40]. Beyond these factors, increasing attention has been given to the role of chrononutrition and meal timing in metabolic regulation [41]. Emerging evidence suggests that the circadian alignment of food intake may significantly influence glucose metabolism, hormonal regulation, inflammatory pathways, and energy homeostasis [42,43]. Disruptions in circadian eating patterns, irregular meal timing, and late-night food consumption have been associated with obesity, insulin resistance, impaired sleep quality, and broader metabolic disturbances [44]. Collectively, these findings support an integrated biopsychosocial model of lifestyle-related health rather than isolated behavioral risk factors.

Given the multidimensional nature of lifestyle-related behaviors, increasing attention has been directed toward psychological mechanisms that facilitate long-term health behavior change. Behavioral self-regulation refers to the capacity to consciously monitor, evaluate, and modify thoughts, emotions, and behaviors in accordance with personally meaningful goals [45]. Higher self-regulatory capacity is associated with healthier dietary patterns, greater physical activity, improved sleep quality, and more effective stress coping [46,47]. In contrast, impaired emotional regulation and reduced self-awareness are linked to maladaptive lifestyle behaviors and poorer psychological and metabolic outcomes [48,49]. Consequently, contemporary lifestyle interventions increasingly focus on enhancing self-awareness and self-regulatory capacity as core mechanisms of sustained health behavior change.

The growing recognition of the relationship between chronic stress, lifestyle behaviors, and health outcomes has also stimulated interest in objective biomarkers capable of capturing long-term physiological stress burden [50]. Among these, hair cortisol concentration (HCC) has emerged as a reliable biomarker of chronic cortisol exposure and HPA-axis activity [51]. Because scalp hair grows approximately 1 cm per month, HCC provides a retrospective assessment of cumulative cortisol secretion over extended periods and is less susceptible to acute fluctuations than serum, salivary, or urinary cortisol measurements [52]. Elevated HCC has been associated with chronic stress, obesity, metabolic syndrome, and adverse health outcomes, supporting its utility as a marker of long-term stress exposure [53,54].

In parallel, telomere biology has emerged as a key framework linking chronic stress, lifestyle behaviors, and biological aging [55]. Telomeres are specialized nucleoprotein structures located at the ends of chromosomes that preserve genomic stability during cellular replication [56]. Progressive telomere shortening occurs naturally with aging but may be accelerated by oxidative stress, inflammation, and chronic activation of stress-related physiological pathways [57]. Consequently, telomere length and other telomere-related biomarkers have gained increasing recognition as indicators of cellular aging and biological resilience. Accelerated telomere attrition has been associated with chronic psychological stress, obesity, metabolic dysfunction, and age-related diseases [58,59,60]. Conversely, healthy lifestyle behaviors—including physical activity, dietary quality, and stress reduction—may support telomere maintenance and cellular resilience, although findings from intervention studies remain heterogeneous.

Despite substantial progress in understanding lifestyle-related health determinants, relatively few intervention studies have simultaneously examined behavioral, psychological, anthropometric, and molecular biomarkers within a unified framework. In particular, evidence remains limited regarding whether interventions targeting self-awareness, emotional regulation, and behavioral self-regulation can induce concurrent changes in subjective outcomes and objective biomarkers of chronic stress and biological aging.

The Pythagorean Self-Awareness Intervention (PSAI) is a structured cognitive-based stress-management approach that combines reflective self-observation, cognitive restructuring, emotional processing, and behavioral self-evaluation. The present intervention is based on the *Golden Verses of Pythagoras*, a collection of 71 short ethical aphorisms traditionally attributed to Pythagoras of Samos and his philosophical school. The *Golden Verses* summarize key principles of Pythagorean philosophy, emphasizing self-discipline, moderation, personal responsibility, and continuous self-examination as foundations for moral and personal development. Within the PSAI, these principles serve as a structured framework for reflective self-appraisal and behavioral self-regulation rather than as a religious or spiritual practice. This philosophical framework is integrated with contemporary cognitive–behavioral and lifestyle medicine principles to support sustainable health-related behavior change, including dietary habits, physical activity, sleep patterns, and interpersonal functioning [61]. By enhancing self-awareness and strengthening behavioral self-regulation, PSAI aims to promote sustainable lifestyle modification, improved stress management, and enhanced psychological well-being. Previous studies have demonstrated beneficial effects on perceived stress, emotional functioning, sleep quality, quality of life, and selected biomarkers of stress and aging [62].

Given the complex interactions between lifestyle behaviors, chronic stress, metabolic regulation, and biological aging, further research is needed to determine whether structured behavioral interventions can produce measurable changes across psychological and biological domains. Therefore, the present study explored whether participation in a six-week behavioral lifestyle intervention incorporating Pythagorean Self-Awareness techniques was associated with changes in dietary habits, anthropometric parameters, psychological functioning, hair cortisol concentration, and biomarkers related to oxidative stress and cellular aging.

## 2. Materials and Methods

### 2.1. Study Design

This study employed a quasi-experimental, single-group pre–post intervention design without a control group. Participants served as their own controls and were assessed at baseline and after completion of the intervention.

A within-subject repeated-measures design was implemented to minimize inter-individual variability and increase sensitivity in detecting changes associated with the intervention. This approach is commonly used in pilot and exploratory behavioral intervention studies involving combined psychological, lifestyle, and biological outcomes, particularly when the primary aim is the preliminary evaluation of multi-domain effects.

Although the absence of a control group limits causal inference, the repeated-measures framework enables the assessment of intra-individual changes over time and improves internal comparability between baseline and post-intervention measurements

### 2.2. Study Setting and Ethical Considerations

The study was conducted at the Medical School of the National and Kapodistrian University of Athens, Athens, Greece. Participant recruitment took place between September and December 2025 in the Attica region.

All participants received detailed written and verbal information regarding the study procedures and provided written informed consent prior to enrolment. The study protocol was approved by the Ethics and Scientific Committee of the First Department of Propaedeutic Internal Medicine, Laiko General Hospital (Protocol No. 2401/27-02-2024) and was conducted in accordance with the Declaration of Helsinki.

### 2.3. Participants and Recruitment

A community-based sample of apparently healthy adults was recruited from the general population of the Attica metropolitan area. Participants were identified through community announcements and local recruitment procedures.

In total, 40 individuals were screened for eligibility, of whom 30 met the inclusion criteria and were enrolled in the study. All enrolled participants completed both baseline and post-intervention assessments and were included in the final analysis.

Eligibility criteria included: (i) age ≥ 18 years, (ii) adequate proficiency in the Greek language, and (iii) permanent residence in the Attica region. Exclusion criteria included current participation in another structured psychological or lifestyle intervention; the presence of diagnosed chronic medical conditions, including diabetes mellitus, cardiovascular disease, autoimmune or chronic inflammatory disorders, active malignancy, severe psychiatric disorders, or any other condition likely to influence oxidative stress, HPA-axis function, or biological aging; and conditions potentially interfering with accurate body composition assessment using bioelectrical impedance analysis (e.g., metallic implants or limb amputation).

All participants provided written informed consent prior to enrolment and completed baseline assessments in accordance with the study protocol.

### 2.4. Intervention: Pythagorean Self-Awareness Intervention (PSAI)

The Pythagorean Self-Awareness Intervention (PSAI) is a structured, evidence-based cognitive–behavioral stress-management intervention grounded in the ethical philosophy of the *Golden Verses of Pythagoras*, a collection of 71 short moral aphorisms traditionally attributed to Pythagoras of Samos (c. 570–495 BCE) and his philosophical school [63,64]. The *Golden Verses* summarize fundamental principles of Pythagorean ethics, emphasizing self-discipline, moderation, personal responsibility, self-reflection, and continuous moral development. Within the PSAI framework, they serve as the philosophical and ethical foundation of the intervention, providing a structured framework for self-examination, conscious self-regulation, and intentional behavioral change rather than functioning as a religious or spiritual practice.

The intervention was delivered over six consecutive weeks and consisted of one face-to-face group-based session per week, each lasting approximately three hours. Sessions were conducted by health professionals experienced in stress management, health promotion, and the PSAI methodology. Each session combined psychoeducational lectures, guided experiential exercises, supervised reflective practice, group discussion, and individualized feedback. Educational topics included stress physiology and stress management, Mediterranean dietary principles, physical activity, sleep hygiene and circadian rhythms, memory and metacognitive processes, cognitive appraisal, emotional regulation, and interpersonal relationships. Diaphragmatic breathing, progressive muscle relaxation, and guided imagery were incorporated as supportive techniques to facilitate concentration and engagement with the reflective process, but they did not constitute the primary therapeutic mechanism of the intervention. Each weekly session followed a standardized structure consisting of (i) review of home practice and participant feedback (approximately 45–60 min), (ii) guided PSAI practice and discussion, and (iii) a psychoeducational module addressing the lifestyle medicine topic scheduled for that week. Participants were encouraged to discuss difficulties encountered during home practice and received individualized feedback from the facilitators.

Participants were instructed to practice the PSAI technique twice daily, once in the morning immediately after awakening and once before bedtime, in a quiet environment free from distractions. The evening practice consisted of five sequential steps: (1) chronological reconstruction of the day’s events; (2) reflection on the thoughts and emotions associated with each event; (3) objective self-observation from the perspective of an impartial internal observer; (4) critical self-appraisal using the three fundamental questions derived from the *Golden Verses* (“What have I done wrong?”, “What have I done right?”, and “What have I omitted that I ought to have done?”); and (5) identification of corrective actions and healthier behavioral alternatives. These questions were not intended to promote moral judgment or self-criticism but to facilitate structured reflection on the consistency between participants’ daily behaviors, their personally endorsed health goals, and the lifestyle principles discussed during the intervention. During the morning practice, participants reviewed the conclusions drawn during the previous evening’s reflection and established realistic behavioral goals for the upcoming day. Throughout the intervention, participants systematically reflected on behaviors related to major lifestyle medicine domains, including dietary habits, physical activity, sleep quality, emotional responses, stress management, and interpersonal relationships.

Adherence was promoted through daily home practice and monitored using attendance records, weekly self-report practice diaries documenting the frequency of PSAI implementation, and structured feedback provided during each group session regarding participants’ experiences, difficulties, and progress in applying the technique. The practical characteristics and implementation protocol of the PSAI are summarized in Table 1 to facilitate reproducibility of the intervention.

The PSAI is designed to cultivate an “internal observer”, enabling individuals to systematically observe, critically evaluate, and consciously regulate their thoughts, emotions, attitudes, and behaviors. Guided by the principles of the *Golden Verses*, participants critically examine their daily experiences, identify maladaptive cognitive and behavioral patterns, recognize discrepancies between their actions and personally endorsed values, and intentionally formulate corrective actions that promote healthier lifestyle choices and psychological well-being. Accordingly, sustainable behavioral change is conceptualized as the outcome of enhanced self-awareness, metacognitive reflection, cognitive restructuring, and ethical self-regulation rather than external instruction alone.

The theoretical model underlying PSAI proposes that repeated cycles of self-observation, reflective self-appraisal, and intentional behavioral planning progressively strengthen metacognitive processes involved in adaptive self-regulation. Rather than relying primarily on external reinforcement or prescriptive behavioral advice, the intervention encourages participants to recognize unhealthy habits, inhibit automatic behavioral responses, and consciously adopt healthier patterns through repeated reflective practice. Thus, lifestyle modification is conceptualized as a self-directed process arising from repeated reflective practice and increasing awareness of one’s own behavior. Rather than classifying thoughts as maladaptive according to predefined diagnostic criteria, participants evaluated whether their thoughts and behaviors were consistent with the ethical principles of the Golden Verses, their personally endorsed health goals, and evidence-based lifestyle recommendations presented during the intervention. Weekly group discussions and facilitator feedback further supported participants in recognizing cognitive and behavioral patterns that interfered with psychological well-being and healthy lifestyle practices. This standardized reflective framework was intended to promote consistency in participants’ self-appraisal across the intervention while allowing individualized reflection based on each participant’s personal experiences and health-related goals.

From a neurocognitive perspective, the reflective component of PSAI is hypothesized to engage neural systems involved in internally directed cognition, particularly the Default Mode Network (DMN). The DMN, comprising interconnected medial prefrontal, posterior cingulate, precuneus, and medial temporal regions, plays a central role in autobiographical memory retrieval, self-referential processing, introspection, future planning, and metacognitive evaluation. During PSAI practice, participants reconstruct daily experiences, reflect on associated thoughts and emotions, and critically evaluate their behaviors from the perspective of the internal observer. These cognitive processes closely correspond to established DMN functions and are hypothesized to facilitate the integration of autobiographical memory with reflective self-processing, thereby strengthening self-awareness, improving emotional regulation, enhancing inhibitory control, and supporting adaptive behavioral regulation.

Overall, PSAI conceptualizes lifestyle modification as a self-directed process emerging from repeated cycles of autobiographical memory retrieval, reflective self-observation, ethical self-appraisal, cognitive restructuring, and intentional behavioral regulation. Within this framework, the philosophical principles of the *Golden Verses of Pythagoras* provide the ethical basis for self-evaluation, whereas contemporary cognitive–behavioral and neuropsychological theories offer a mechanistic explanation for how repeated reflective practice may facilitate enduring improvements in health-related behaviors and psychological well-being.

### 2.5. Outcome Measures

Participants’ demographic characteristics included age, marital status, educational level, occupational status, health status, medication use, alcohol intake, and smoking habits. Clinical, anthropometric, physiological, psychological, and biological data were collected at baseline and post-intervention.

#### 2.5.1. Anthropometric and Body Composition Measures

Body weight and height were measured using standardized procedures with participants wearing light clothing and no shoes. Body composition was assessed using multi-frequency bioelectrical impedance analysis (BIA-ACC device, BIOTEKNA ©, Venice, Italy), previously validated against dual-energy X-ray absorptiometry (DXA).

Outcomes included body mass index (BMI), fat-free mass (FFM), and intermuscular adipose tissue (IMAT).

#### 2.5.2. Dietary Assessment

Adherence to the Mediterranean dietary pattern was assessed using the validated MedDiet Score (range 0–56). The instrument evaluates frequency of consumption of food groups characteristic of the Mediterranean diet, including fruits, vegetables, legumes, whole grains, olive oil, fish, and limited intake of red and processed meat. Higher scores indicate greater adherence [65].

#### 2.5.3. Psychological and Behavioral Measures

All psychological instruments used validated Greek versions and were administered according to standardized scoring guidelines.

Perceived Stress Scale (PSS-14)

The PSS-14 is a widely used 14-item questionnaire assessing the degree to which individuals perceive life situations as stressful over the past month. Items are rated on a 5-point Likert scale, with higher scores indicating higher perceived stress. The scale includes both positively and negatively phrased items to reduce response bias [66]

Depression Anxiety Stress Scales (DASS-21)

The DASS-21 is a 21-item instrument measuring emotional distress across three domains: depression, anxiety, and stress. Each subscale consists of seven items assessing symptom severity over the previous week. Higher scores indicate greater psychological distress [67].

Rosenberg Self-Esteem Scale (RSES)

The RSES is a 10-item measure of global self-esteem and self-worth. Items are rated on a 4-point Likert scale, with higher scores reflecting greater self-esteem [68].

Healthy Lifestyle and Personal Control Questionnaire (HLPCQ)

The HLPCQ is a 26-item instrument assessing health-promoting behaviors and perceived behavioral self-regulation. It evaluates domains including dietary habits, physical activity, daily routine organization, and psychological well-being. Higher scores indicate stronger engagement in health-promoting behaviors and greater perceived control over lifestyle choices [69].

Health Locus of Control Scale (HLC)

The HLC is an 18-item questionnaire assessing beliefs regarding health control. It includes three subscales: internal control, external control, and chance control. Higher scores reflect stronger endorsement of each dimension [70].

State–Trait Anger Expression Inventory (STAXI)

The STAXI assesses anger experience and expression, including anger-in, anger-out, and anger control dimensions. Items are rated on a 4-point Likert scale, with higher scores indicating stronger expression or regulation tendencies [71].

State Shame and Guilt Scale (SSGS)

The SSGS is a 15-item scale assessing current emotional states of shame, guilt, and pride. Participants rate intensity on a 5-point Likert scale [72].

Pittsburgh Sleep Quality Index (PSQI)

The PSQI is a 19-item questionnaire assessing sleep quality over the past month across seven domains, including sleep latency, duration, efficiency, disturbances, and daytime dysfunction. Global scores range from 0 to 21, with higher scores indicating poorer sleep quality [73].

#### 2.5.4. Cognitive Measures

Cognitive function was assessed using the Brief International Cognitive Assessment for Multiple Sclerosis (BICAMS), which includes the Brief Visuospatial Memory Test–Revised (BVMT-R), the California Verbal Learning Test–II (CVLT-II), and the Symbol Digit Modalities Test (SDMT). The battery evaluates visuospatial memory, verbal learning, and information processing speed, respectively, and has been previously validated in the Greek population [74]. BICAMS was selected as a standardized and validated measure of cognitive performance because its assessment of information processing speed and memory is relevant to the cognitive processes engaged during PSAI practice. In the present study, cognitive outcomes were considered exploratory and were included to examine whether participation in the intervention was associated with concurrent changes in cognitive functioning.

#### 2.5.5. Biological Measures

Hair Cortisol Concentration

Hair cortisol concentration (HCC) was used as a biomarker of long-term hypothalamic–pituitary–adrenal (HPA) axis activity. Hair samples were collected from the posterior vertex region, which is the recommended anatomical site for hair cortisol assessment because of its relatively uniform growth characteristics and lower intra-individual variability compared with other scalp regions, and the proximal 1 cm segment was analyzed, reflecting approximately one month of cumulative cortisol secretion [75].

Leukocyte Telomere Length

Leukocyte telomere length was assessed as a marker of cellular aging. Blood samples (2–4 mL) were collected in EDTA tubes, processed within 4 h, and stored at −20 °C until analysis.

Genomic DNA was extracted using the NucleoSpin Blood Kit (Macherey-Nagel, Düren, Germany). Relative telomere length was quantified using quantitative PCR (qPCR) on a Roche LightCycler 480 system, measuring telomere repeat copy number (T) and a single-copy gene (albumin, S). All samples were analyzed in triplicate. A standard curve based on serial dilutions was used to ensure amplification efficiency. Results were expressed as the T/S ratio and normalized using z-scores to correct for inter-batch variability.

Serum Oxidative Stress and Antioxidant Capacity

Blood samples were centrifuged at 4000 rpm for 10 min and serum was stored at −80 °C until analysis. All assays were performed in duplicate.

Serum oxidative stress was measured using a Total Oxidant Status (TOS) ELISA kit (EEA027, Thermo Fisher Scientific, Waltham, MA, USA) according to manufacturer instructions.

Total antioxidant capacity was assessed using a DPPH radical scavenging assay. Serum samples were deproteinized with Proteinase K, incubated with methanolic DPPH solution, and absorbance was measured at 517 nm. Results were expressed as percentage radical scavenging activity relative to Trolox standards.

### 2.6. Data Collection

Data were collected between September and December 2025 at the Medical School of the National and Kapodistrian University of Athens. Following written informed consent, participants underwent baseline assessments prior to the initiation of the PSAI.

Assessments were repeated after completion of the six-week intervention. Data collection included anthropometric and body composition measurements, validated psychometric questionnaires, hair cortisol sampling, and blood sampling for biochemical and molecular analyses.

### 2.7. Statistical Analysis

Statistical analyses were performed using IBM SPSS Statistics for Windows, version 25.0 (IBM Corp., Armonk, NY, USA). The normality of continuous variables was assessed using the Shapiro–Wilk test. Descriptive statistics are presented as mean ± standard deviation (SD) or median and interquartile range (IQR), as appropriate.

Changes between baseline and post-intervention measurements were evaluated using paired-samples *t*-tests for normally distributed variables and Wilcoxon signed-rank tests for non-normally distributed variables. Effect sizes were interpreted according to conventional criteria. Cohen’s *d* values of 0.20, 0.50, and 0.80 were considered small, medium, and large effects, respectively, whereas *r* values of 0.10, 0.30, and 0.50 were interpreted as small, medium, and large effects.

To account for multiple comparisons across the 31 predefined outcomes, the Benjamini–Hochberg procedure was applied to control the false discovery rate at 5%. Both unadjusted and BH-FDR-adjusted *p*-values are reported, with statistical significance after correction defined as a BH-FDR-adjusted *p*-value < 0.05.

Given the exploratory nature of this pilot study, no a priori sample size calculation was performed. The study was intended to identify preliminary within-subject changes and generate effect-size estimates for future randomized controlled trials. Accordingly, the findings should be interpreted as hypothesis-generating despite adjustment for multiple comparisons.

## 3. Results

### 3.1. Participants’ Characteristics

Table 2 presents the sociodemographic characteristics of the 30 participants. The study sample consisted of 30 apparently healthy adults, with a slight predominance of females (56.7%) over males (43.3%). The median age of the participants was 52.5 years (IQR = 23).

Most participants were married (63.3%) and employed (80.0%). Regarding educational level, more than half of the sample had completed post-secondary or tertiary education (56.7%), while 30.0% held a postgraduate qualification (MSc/PhD). The majority of participants lived in urban areas (83.3%) and reported cohabiting with others (80%). In terms of family status, 60.0% reported having children.

Regarding lifestyle-related characteristics, 40% of participants were current smokers, while 26.7% were former smokers and 33.3% were non-smokers. Alcohol consumption was reported by 36.7% of the sample.

### 3.2. Anthropometric Characteristics and Mediterranean Diet Adherence

Table 3 presents the changes in anthropometric characteristics and Mediterranean diet adherence before and after the intervention. A statistically significant reduction was observed in body mass index (BMI) following the intervention (*p* = 0.012), accompanied by a corresponding decrease in body weight (*p* = 0.012). Significant reductions were also observed in neck circumference (*p* = 0.012), waist circumference (*p* = 0.007), and hip circumference (*p* = 0.002).

Body composition analysis indicated a significant decrease in intermuscular adipose tissue (IMAT) (*p* = 0.012), while fat-free mass (FFM) and basal metabolic rate (BMR) showed non-significant trends toward change (*p* = 0.072 and *p* = 0.077, respectively). Total body water distribution showed significant changes, with an increase in intracellular water (ICW) (*p* = 0.043) and a decrease in extracellular water (ECW) (*p* = 0.027). Overall body water percentage also showed a significant increase (*p* = 0.006).

Mediterranean diet adherence improved significantly following the intervention, as indicated by an increase in the total MedDiet score (*p* = 0.001).

All anthropometric and dietary findings that were statistically significant in the primary analyses remained significant following BH-FDR correction. The adjusted results for intracellular water were close to the significance threshold (BH-FDR-adjusted *p* = 0.049), whereas fat-free mass and basal metabolic rate remained non-significant.

### 3.3. Changes in Psychological and Behavioral Outcomes

Table 4 presents changes in psychological and behavioral outcomes following the intervention. Significant improvements were observed in healthy lifestyle behaviors and perceived personal control, as reflected by higher HLPCQ scores at post-intervention assessment compared with baseline (*p* < 0.001).

Regarding health-related beliefs, participants demonstrated a significant increase in internal health locus of control (*p* = 0.002) and a significant decrease in external health locus of control (*p* = 0.015), whereas chance health locus of control remained unchanged (*p* = 0.285).

Significant reductions were observed in psychological distress. Specifically, DASS-Stress (*p* = 0.035), DASS-Depression (*p* = 0.005), and perceived stress measured by the PSS (*p* = 0.004) all decreased significantly following the intervention. In addition, participants reported lower levels of shame (*p* = 0.013) and guilt (*p* = 0.036), together with higher levels of pride (*p* = 0.014), as assessed by the SSGS.

Further improvements were observed in emotional regulation and self-perception. STAXI scores decreased significantly (*p* = 0.003), indicating lower anger expression, while self-esteem, assessed by the RSES, increased significantly following the intervention (*p* = 0.003).

Finally, sleep quality improved significantly, as evidenced by lower PSQI scores at post-intervention assessment compared with baseline (*p* < 0.001).

Overall, the intervention was associated with significant improvements across multiple domains, including perceived stress, depressive symptoms, health-related beliefs, self-conscious emotions, anger regulation, self-esteem, lifestyle behaviors, and sleep quality. All reported psychological and behavioral changes, with the exception of chance health locus of control, remained statistically significant after BH-FDR correction. The adjusted results for DASS-Stress and SSGS-Guilt approached the significance threshold (both BH-FDR-adjusted *p* = 0.045).

### 3.4. Changes in Cognitive Performance

Table 5 presents changes in cognitive performance following the intervention. Significant improvements were observed across all cognitive domains assessed. Information processing speed, measured using the SDMT, increased significantly following the intervention (*p* = 0.014). Similarly, verbal memory performance, assessed with the CVLT-II, improved significantly between baseline and post-intervention assessments (*p* = 0.012).

Visuospatial memory, evaluated using the BVMT-R, also demonstrated a significant improvement following the intervention (*p* = 0.042). Effect sizes for the observed changes were small to moderate, suggesting meaningful improvements in cognitive performance across multiple domains.

Overall, participation in the intervention was associated with enhanced information processing speed, verbal memory, and visuospatial memory. All three cognitive outcomes remained statistically significant following BH-FDR correction, although the adjusted result for visuospatial memory was close to the significance threshold (BH-FDR-adjusted *p* = 0.049).

### 3.5. Effects of the Intervention on Biological Markers

Table 6 presents changes in biological markers following the intervention. A significant reduction in oxidative stress levels was observed, with median values decreasing from 13.99 (IQR: 12.45–16.87) at baseline to 12.46 (IQR: 11.34–14.11) post-intervention (*p* = 0.023, r = 0.45). In parallel, antioxidant capacity increased significantly from 537.50 (IQR: 456.75–598.50) to 550.00 (IQR: 526.00–645.50) following the intervention (*p* = 0.009, r = 0.52).

Leukocyte telomere length increased significantly from 1.49 ± 0.39 to 2.03 ± 0.54 (*p* < 0.001), corresponding to a large effect size (Cohen’s d = 1.53). In contrast, no statistically significant change was observed in hair cortisol concentration, which increased from 10.91 ± 6.17 to 12.18 ± 6.18 following the intervention (*p* = 0.102, d = 0.26).

**Figure 1:**
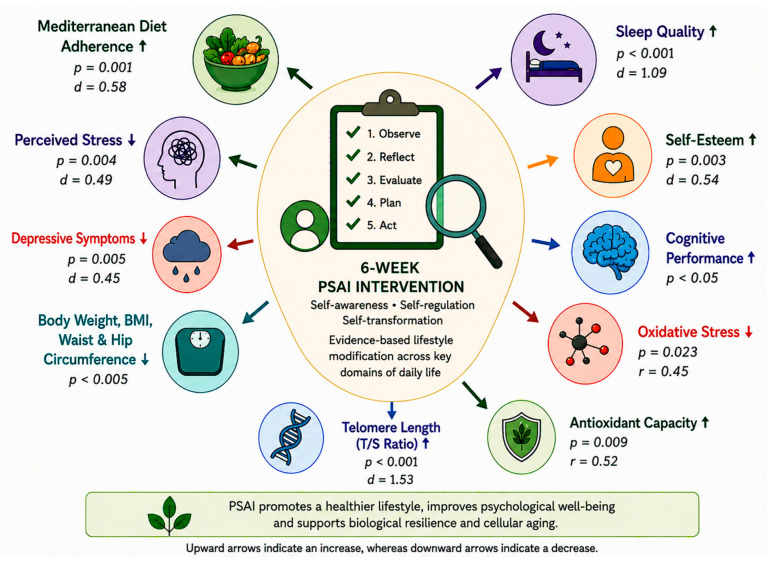
Key Outcomes of the 6-week PSAI.

Overall, the intervention was associated with improvements in biomarkers related to oxidative balance and cellular aging, whereas no significant changes were observed in hair cortisol concentration. The reductions in oxidative stress and increases in antioxidant capacity and relative leukocyte telomere length remained statistically significant following BH-FDR correction, whereas hair cortisol concentration remained non-significant. The principal clinical, physiological, and lifestyle outcomes of the 6-week PSAI are illustrated in Figure 1.

## 4. Discussion

The present study explored the associations of a six-week holistic behavioral intervention based on the Pythagorean Self-Awareness Intervention (PSAI) with lifestyle behaviors, psychological functioning, cognitive performance, anthropometric outcomes, oxidative stress biomarkers, and markers of biological aging in apparently healthy adults. Overall, participation in the intervention was associated with favorable changes across multiple domains, including greater adherence to the Mediterranean diet, healthier lifestyle behaviors, improved sleep quality, reduced perceived stress and depressive symptoms, enhanced emotional regulation and self-esteem, modest improvements in anthropometric parameters, reduced oxidative stress, increased antioxidant capacity, and an increase in relative leukocyte telomere length. In contrast, no significant changes were observed in hair cortisol concentration.

Taken together, these findings provide preliminary evidence that a structured self-awareness-based intervention may be associated with multidimensional improvements in behavioral, psychological, and physiological health. However, given the quasi-experimental single-group design, the findings should be interpreted cautiously as hypothesis-generating rather than confirmatory. Within this context, the results are discussed in relation to current evidence from lifestyle medicine, psychoneuroendocrinology, and behavioral science.

### 4.1. Interpretation of Findings in Relation to Lifestyle Behavior Change Models

A central finding of the present study was the significant improvement in multiple lifestyle-related behaviors, particularly Mediterranean diet adherence, sleep quality, and health-promoting behaviors. These results are consistent with the hypothesis that enhanced self-awareness and behavioral self-regulation can facilitate sustainable lifestyle modification [76]. Previous studies have shown that structured lifestyle interventions improve dietary quality and overall health behaviors, particularly when they incorporate self-monitoring and reflective practices [77,78,79,80]. However, most conventional interventions rely primarily on prescriptive dietary or exercise recommendations [81]. In contrast, PSAI emphasized systematic self-observation and reflective evaluation of daily behaviors rather than externally imposed behavioral rules, suggesting that cognitive–behavioral restructuring may facilitate meaningful lifestyle change.

This interpretation is consistent with self-regulation theory, which proposes that awareness of one’s own behavior is a prerequisite for sustained behavior modification [82]. By encouraging participants to reconstruct daily experiences and evaluate their actions from an observer perspective, PSAI may promote the recognition of maladaptive habits and support autonomous lifestyle modification across multiple behavioral domains.

The observed improvement in Mediterranean diet adherence further supports this interpretation. Extensive evidence has linked the Mediterranean dietary pattern with reduced cardiometabolic risk, lower systemic inflammation, and improved psychological well-being [83,84,85,86,87,88,89]. In the present study, however, dietary improvements appeared to emerge through enhanced reflective awareness rather than structured nutritional counseling, suggesting that self-awareness may represent an additional mechanism supporting healthier dietary choices. Although Greece is traditionally regarded as the birthplace of the Mediterranean dietary pattern, adherence among contemporary Greek adults has declined substantially over recent decades as dietary habits have progressively shifted toward a more westernized pattern. Accordingly, the moderate baseline Mediterranean Diet Score observed in the present sample indicates that considerable scope for dietary improvement remained despite the Mediterranean setting. This context may partly explain the significant improvement in adherence observed following the intervention.

### 4.2. Anthropometric Changes and Metabolic Implications

Significant reductions were observed in body weight, BMI, waist circumference, hip circumference, and intermuscular adipose tissue. Although fat-free mass and basal metabolic rate did not change significantly, the overall pattern suggests favorable metabolic adaptation.

These findings are particularly relevant because they were achieved without a formal calorie-restricted diet. This supports the hypothesis that behavioral awareness alone may influence energy balance through subtle changes in eating behavior, self-regulation, and daily decision-making [90,91]. Similar findings have been reported in mindfulness-based and cognitive–behavioral interventions, where improvements in eating awareness and emotional regulation lead to modest but meaningful weight-related outcomes [92,93].

From a mechanistic perspective, these changes may reflect improved interoceptive awareness and reduced emotional eating [94]. Chronic stress is known to influence appetite regulation through cortisol-mediated pathways, often promoting increased caloric intake and preference for energy-dense foods [95,96]. By reducing perceived stress and enhancing emotional regulation, PSAI may indirectly contribute to improved metabolic outcomes.

### 4.3. Sleep Quality as a Key Mediator of Health Improvements

The significant improvement in sleep quality observed in the present study is consistent with the growing recognition of sleep as a central determinant of metabolic, cognitive, and emotional health [97]. Sleep disruption has been strongly associated with obesity, insulin resistance, cardiovascular disease, and impaired psychological functioning [98,99]. The PSAI incorporates multiple components known to influence sleep physiology, including diaphragmatic breathing, progressive muscle relaxation, guided imagery, and cognitive reflection. These techniques may reduce autonomic hyperarousal and cognitive rumination, both of which are strongly implicated in sleep disturbances.

Previous studies have shown that mindfulness-based and stress-reduction interventions can improve sleep quality by modulating sympathetic nervous system activity and reducing cognitive–emotional arousal [100,101]. The present findings extend this literature by suggesting that structured self-awareness practices may similarly enhance sleep quality, potentially through both physiological relaxation and cognitive restructuring mechanisms.

Importantly, sleep improvements may also act as a mediating pathway through which other outcomes, such as cognitive performance and metabolic regulation, are improved.

### 4.4. Psychological Mechanisms and Emotional Regulation

The intervention produced significant improvements in perceived stress, depressive symptoms, anger expression, self-esteem, and self-conscious emotions. These findings strongly support the theoretical framework of PSAI, which is grounded in reflective awareness, cognitive restructuring, and emotional processing. The reduction in stress and depressive symptoms is consistent with a large body of evidence demonstrating that chronic stress negatively affects emotional regulation and psychological resilience [102]. Conversely, interventions that enhance self-awareness and cognitive reframing have been shown to reduce psychological distress and improve adaptive coping strategies [103].

A key interpretation is that PSAI enhances metacognitive awareness, allowing individuals to observe their thoughts and behaviors without immediate emotional reactivity. This process may reduce maladaptive cognitive patterns such as rumination, self-criticism, and emotional suppression, thereby improving overall emotional balance.

Importantly, improvements in psychological functioning likely contributed to behavioral changes observed in diet, sleep, and lifestyle behaviors [104]. This bidirectional relationship between psychological and behavioral domains is consistent with contemporary biopsychosocial models of health behavior change.

### 4.5. Cognitive Performance and Potential Neurocognitive Mechanisms

Although cognitive outcomes were considered exploratory in the present study, improvements were observed in information processing speed, verbal memory, and visuospatial memory. These findings are consistent with the cognitive demands inherent in PSAI, which requires repeated autobiographical memory retrieval, temporal sequencing, reflective self-appraisal, and metacognitive processing.

Participants were required to engage in structured recall of daily events, temporal sequencing, behavioral evaluation, and reflective analysis. These processes engage multiple cognitive domains, including working memory, episodic memory retrieval, attention, and executive functioning. Therefore, PSAI may function as a form of metacognitive training that strengthens higher-order cognitive processes.

In addition, reductions in stress and improvements in sleep quality may have contributed to enhanced cognitive performance [105]. Chronic stress is known to impair hippocampal-dependent memory processes [106], while sleep plays a crucial role in memory consolidation and cognitive restoration [107]. Therefore, the observed cognitive improvements may reflect both direct cognitive engagement and indirect physiological benefits.

### 4.6. Oxidative Stress, Antioxidant Capacity, and Biological Aging

At the biological level, the intervention was associated with a significant reduction in oxidative stress and an increase in antioxidant capacity, indicating an improved redox balance. These findings are consistent with the hypothesis that behavioral and psychological interventions can influence systemic physiological processes.

Chronic psychological stress is known to increase oxidative stress through glucocorticoid-mediated pathways and mitochondrial dysfunction [108,109]. By reducing perceived stress and improving behavioral regulation, PSAI may indirectly reduce oxidative load and enhance antioxidant defenses.

A particularly noteworthy finding was the observed increase in relative leukocyte telomere length as assessed by qPCR. Although statistically significant, this finding should be interpreted cautiously because qPCR-derived T/S ratios are relative measures and may be influenced by analytical variability, differences in leukocyte subpopulations, and short-term biological fluctuations. Although some intervention studies have reported favorable changes in relative telomere length following intensive lifestyle modification, the magnitude of change observed in the present study exceeds what would generally be expected over a six-week period. Consequently, these findings should not be interpreted as definitive evidence of rapid telomere elongation but rather as preliminary observations requiring confirmation using larger samples, repeated measurements, and complementary methodologies with high analytical precision. Telomeres are widely recognized as biomarkers of cellular aging, and their shortening is accelerated by stress, inflammation, and oxidative damage [110].

Previous studies have linked lifestyle factors such as diet quality, physical activity, stress reduction, and sleep quality to telomere maintenance [111,112,113,114,115,116]. The present findings extend this literature by suggesting that a structured self-awareness-based intervention may simultaneously influence multiple behavioral pathways associated with telomere biology.

Although the exact mechanisms remain unclear, reductions in oxidative stress, improved emotional regulation, and enhanced lifestyle behaviors likely contributed synergistically to improved telomere dynamics.

The multidomain nature of the intervention may partly explain the magnitude of the observed change. Unlike interventions targeting a single lifestyle component, PSAI simultaneously addresses several core domains of lifestyle medicine, including dietary habits, stress management, sleep, physical activity, and behavioral self-regulation. The concurrent modification of these interrelated lifestyle factors may have exerted synergistic effects on biological pathways involved in oxidative stress regulation and cellular aging.

Nevertheless, the observed magnitude of change should be interpreted cautiously. Relative telomere length assessed by qPCR may be influenced by technical variability, leukocyte population shifts, and short-term biological fluctuations. Therefore, although the findings are encouraging, replication in larger controlled studies using multiple assessments and longer follow-up periods is warranted before definitive conclusions regarding telomere dynamics can be drawn.

### 4.7. Dissociation Between Psychological Improvements and HPA-Axis Activity

Despite broad improvements across psychological and behavioral outcomes, no significant changes were observed in hair cortisol concentration. This dissociation suggests that subjective and behavioral improvements may precede measurable changes in long-term hypothalamic–pituitary–adrenal (HPA) axis activity.

Hair cortisol reflects cumulative cortisol secretion over extended periods [117] and may require longer intervention durations to show detectable changes. Additionally, HPA-axis regulation is influenced by complex neuroendocrine feedback loops that may not respond rapidly to behavioral interventions.

This finding is consistent with previous research showing temporal mismatches between psychological stress reduction and endocrine biomarkers [118,119]. It suggests that improvements in perceived stress and emotional well-being may represent earlier stages of adaptation, while physiological recalibration may occur over longer timescales.

### 4.8. Integrative Interpretation of the Findings

Taken together, the findings support a biopsychosocial model in which behavioral self-awareness acts as a central mechanism linking psychological processes with physiological and molecular outcomes. The PSAI appears to influence health through multiple interconnected pathways, including cognitive restructuring, emotional regulation, behavioral modification, and stress reduction.

This integrative model is consistent with contemporary perspectives in lifestyle medicine and psychoneuroimmunology, which emphasize the bidirectional interaction between mind and body systems [120].

### 4.9. Clinical Relevance

Although the observed effect sizes should be interpreted cautiously due to the pilot nature of the study, the findings suggest that structured self-awareness-based interventions may represent a feasible and low-cost adjunctive approach for promoting healthy lifestyle behaviors and psychological well-being in community populations. If confirmed in randomized controlled trials, such interventions could complement existing lifestyle medicine strategies by targeting self-regulation processes that underlie long-term behavior change.

Although the present findings suggest that self-awareness-based interventions may facilitate healthier lifestyle choices, successful implementation also depends on broader contextual factors. Economic and environmental constraints, food accessibility, occupational demands, neighborhood environments, and opportunities for physical activity may substantially influence an individual’s ability to adopt and sustain healthy behaviors. Therefore, interventions such as PSAI should be viewed as complementary to, rather than substitutes for, broader public health strategies addressing the social determinants of health.

### 4.10. Strengths, Limitations, and Methodological Considerations

The present study has several strengths, including its multidimensional assessment of behavioral, psychological, anthropometric, and biological outcomes, the integration of subjective and objective measures, and the evaluation of biomarkers related to oxidative stress and biological aging. In addition, the standardized PSAI protocol enhances the reproducibility of the intervention and supports its potential applicability within lifestyle medicine.

Several limitations should also be acknowledged. First, the single-group quasi-experimental design without a control group precludes causal inference, and alternative explanations for the observed changes, including regression to the mean, expectancy (placebo) effects, and seasonal influences, cannot be excluded. Second, the relatively small sample size and the predominance of female participants limit the generalizability of the findings. Third, the use of self-report questionnaires may have introduced reporting bias, while the six-week intervention period may have been insufficient to detect longer-term biological adaptations, particularly with respect to HPA-axis regulation and cellular aging markers. Smoking was not an exclusion criterion, and 40% of the sample consisted of current smokers. Because smoking and nicotine exposure may affect HPA-axis activity, oxidative stress, and telomere biology, residual confounding by smoking status cannot be excluded. The limited sample size did not permit adequately powered stratified or adjusted analyses according to smoking status.

Multiple statistical comparisons were performed across the predefined outcomes. To reduce the risk of false-positive findings, the Benjamini–Hochberg procedure was applied to control the false discovery rate at 5%. All outcomes that were statistically significant in the primary analyses remained significant after correction, although several adjusted results were close to the significance threshold. Nevertheless, because of the limited sample size and exploratory design, the findings should still be interpreted as preliminary and hypothesis-generating and require confirmation in larger, adequately powered randomized controlled trials.

### 4.11. Future Research Directions

Future studies should aim to replicate these findings in larger randomized controlled trials with appropriate control conditions. Longer follow-up periods are essential to determine the sustainability of behavioral and biological changes, particularly regarding telomere dynamics and cortisol regulation. Additionally, future research should investigate mechanistic pathways linking self-awareness, stress reduction, and biological aging, potentially incorporating inflammatory markers, epigenetic measures, and neuroimaging techniques. It would also be valuable to compare PSAI with other mindfulness-based and cognitive–behavioral interventions to determine its relative efficacy and specificity. Finally, mediation analyses could help clarify whether improvements in psychological functioning mediate changes in lifestyle behaviors and biological outcomes, providing deeper insight into causal pathways.

## 5. Conclusions

This study indicates that a six-week Pythagorean Self-Awareness Intervention (PSAI) may induce multi-domain improvements in apparently healthy adults, encompassing lifestyle behaviors, psychological functioning, cognition, and biomarkers of oxidative stress and biological aging. Participants showed improved Mediterranean diet adherence, health-promoting behaviors, sleep quality, and favorable changes in anthropometric indices.

These behavioral changes were accompanied by reductions in perceived stress, depressive symptoms, anger-related outcomes, and negative self-conscious emotions, alongside increases in self-esteem and adaptive emotional states. Cognitive performance improved in processing speed and memory domains. At the biological level, oxidative stress decreased, while an increase in relative leukocyte telomere length was observed, whereas hair cortisol concentration remained unchanged.

Collectively, the findings support the potential of structured self-awareness-based interventions to influence behavioral, psychological, and biological pathways relevant to health and aging. However, the absence of a control group, the relatively small sample size, and the short intervention duration limit the ability to draw firm causal conclusions.

These findings provide preliminary evidence supporting the potential value of the Pythagorean Self-Awareness Intervention across multiple domains. Nevertheless, because of the quasi-experimental single-group design, the results should be interpreted with appropriate caution and confirmed in larger randomized controlled studies before firm conclusions regarding efficacy can be drawn.

## Figures and Tables

**Table 1:** Operational characteristics and implementation protocol of the Pythagorean Self-Awareness Intervention (PSAI).

Component	Description
Study duration	Six consecutive weeks
Session frequency	One face-to-face group session per week
Session duration	Approximately 3 h per session
Facilitators	The intervention was delivered by health professionals trained in the PSAI methodology with expertise in stress management and health promotion
Intervention format	Group-based psychoeducational and experiential intervention combining theoretical instruction, guided reflective practice, group discussion, and individualized feedback
Educational content	Stress physiology and stress management; Mediterranean dietary principles; physical activity; sleep hygiene and circadian rhythms; memory and metacognitive processes; cognitive appraisal; emotional regulation; interpersonal relationships
Supportive techniques	Diaphragmatic breathing, progressive muscle relaxation, and guided imagery were used to facilitate concentration and engagement but were not considered the primary therapeutic components of the intervention
Daily home practice	Participants practiced the PSAI technique twice daily (morning and evening) throughout the intervention period
Core PSAI procedure	Step 1: Sequential recall of daily events in chronological order. Step 2: Reflection on the thoughts and emotions associated with each event. Step 3: Objective self-observation from the perspective of an impartial internal observer. Step 4: Critical self-appraisal using the three reflective questions derived from the *Golden Verses*: *What have I done wrong?*, *What have I done right?*, and *What have I omitted that I ought to have done?* Step 5: Morning review of the previous evening’s reflection and establishment of realistic behavioral goals for the upcoming day
Lifestyle domains addressed	Dietary habits, physical activity, sleep quality, stress management, emotional regulation, interpersonal relationships, and other health-related behaviors
Session structure	Each session included: (i) review of participants’ experiences and home practice, (ii) guided reflective practice and discussion, (iii) psychoeducational lecture on the weekly lifestyle medicine topic, and (iv) individualized feedback and behavioral goal setting
Adherence monitoring	Attendance records, weekly self-report practice diaries documenting home practice frequency, and facilitator feedback during weekly sessions

**Table 2:** Sociodemographic characteristics of the study participants.

Variable	Category	*n* (%)
Sex	Male	13 (43.33)
	Female	17 (56.67)
Marital status	Married	19 (63.3)
	Single	7 (23.3)
	Divorced	3 (10.0)
	Widowed	1 (3.3)
Education level	Until Upper Secondary School	4 (13.3)
	Post-Secondary/Tertiary	17 (56.7)
	MSc/PhD	9 (30.0)
Employment status	Employed	24 (80.0)
	Unemployed	3 (10.0)
	Retired	3 (10.0)
Living arrangement	Cohabiting	24 (80.0)
	Living alone	6 (20.0)
Place of residence	Urban	25 (83.3)
	Rural	5 (16.7)
Having children	Yes	18 (60.0)
	No	12 (40.0)
Smoking status	Current smoker	12 (40.0)
	Non-smoker	10 (33.3)
	Former smoker	8 (26.7)
Alcohol consumption	Yes	11 (36.7)
	No	19 (63.3)

**Table 3:** Changes in anthropometric characteristics and Mediterranean diet adherence before and after the intervention.

Variable *	Pre-Intervention Mean (SD)	Post-Intervention Mean (SD)	Effect Size (r/Cohen’s d)	*p*-Value	BH-FDR-Adjusted *p*-Value
BMI	24.62 (4.30)	24.12 (4.12)	−0.42	0.012 *	0.022 *
WEIGHT	70.24 (13.81)	69.01 (13.75)	−0.42	0.012 *	0.022 *
FFM	47.30 (9.12)	47.50 (9.38)	0.30	0.072	0.080
BMR	1391 (197.50)	1396 (203)	0.30	0.077	0.082
IMAT	1.45 (1)	1.30 (0.7)	−0.40	0.012 *	0.022 *
NC	33.91 (4.24)	32.91 (3.82)	−0.41	0.012 *	0.022 *
WC	84.79 (12.47)	82.36 (11.95)	−0.43	0.007 *	0.020 *
HC	100.33 (9.59)	98.19 (10.65)	−0.50	0.002 *	0.010 *
ICW	17.02 (6.39)	18.26 (7.23)	0.32	0.043 *	0.049 *
ECW	49.38 (4.90)	47.50 (6.02)	0.36	0.027 *	0.036 *
MDTOTAL	27.00 (5.07)	29.41 (4.20)	0.58	0.001 *	0.008 *

* BMI: Body Mass Index; FFM: Fat-Free Mass; BMR: Basal Metabolic Rate; IMAT: Intermuscular Adipose Tissue; NC: Neck Circumference; WC: Waist Circumference; HC: Hip Circumference; ICW: Intracellular Water; ECW: Extracellular Water; MDTOTAL: Mediterranean Diet Total Score.

**Table 4:** Changes in psychological and behavioral outcomes following the intervention.

Variable *	Pre-Intervention Mean (SD)	Post-Intervention Mean (SD)	Effect Size (r/Cohen’s d)	*p*-Value	BH-FDR-Adjusted *p*-Value
HLPCQ Total Score	67.15 (9.44)	72.51 (9.50)	0.74	<0.001 *	0.008 *
HLC Internal	27.35 (5.71)	29.28 (4.12)	0.49	0.002 *	0.010 *
HLC Chance	18.00 (6.90)	18.00 (6.90)	0.23	0.285	0.285
HLC External	14.00 (6.02)	11.00 (5.99)	0.39	0.015 *	0.022 *
DASS-Stress	10.00 (8.39)	8.00 (6.59)	0.35	0.035 *	0.045 *
DASS-Depression	4.00 (8.63)	2.00 (5.01)	0.45	0.005 *	0.016 *
SSGS-Shame	6.00 (4.33)	5.00 (3.19)	0.40	0.013 *	0.022 *
SSGS-Guilt	11.00 (4.97)	10.00 (4.24)	0.34	0.036 *	0.045 *
SSGS-Pride	22.00 (4.96)	23.00 (3.78)	0.39	0.014 *	0.022 *
PSS-14	24.00 (8.09)	19.00 (7.22)	0.49	0.004 *	0.014 *
STAXI	22.17 (7.56)	18.74 (6.87)	0.52	0.003 *	0.012 *
RSES	30.94 (5.44)	32.76 (5.36)	0.54	0.003 *	0.012 *
PSQI	6.00 (2.87)	5.00 (1.87)	1.09	<0.001 *	0.008 *

* HLPCQ, Healthy Lifestyle and Personal Control Questionnaire; HLC, Health Locus of Control Scale; DASS, Depression Anxiety Stress Scales; SSGS, State Shame and Guilt Scale; PSS, Perceived Stress Scale; STAXI, State–Trait Anger Expression Inventory; RSES, Rosenberg Self-Esteem Scale; PSQI, Pittsburgh Sleep Quality Index.

**Table 5:** Changes in cognitive performance following the intervention.

Variable *	Pre-Intervention Mean (SD)	Post-Intervention Mean (SD)	Effect Size (r/Cohen’s d)	*p*-Value	BH-FDR-Adjusted *p*-Value
SDMT (Information Processing Speed)	50.00 (15.30)	57.00 (14.24)	0.41	0.014 *	0.022 *
CVLT-II (Verbal Memory)	73.00 (7.66)	74.00 (5.69)	0.40	0.012 *	0.022 *
BVMT-R (Visuospatial Memory)	30.00 (9.50)	32.00 (5.50)	0.38	0.042 *	0.049 *

* SDMT, Symbol Digit Modalities Test; CVLT-II, California Verbal Learning Test—Second Edition; BVMT-R, Brief Visuospatial Memory Test–Revised.

**Table 6:** Changes in biological markers following the intervention.

Variable	Pre-Intervention Mean (SD)	Post-Intervention Mean (SD)	Effect Size (r/Cohen’s d)	*p*-Value	BH-FDR-Adjusted *p*-Value
Oxidative stress *	13.99 (12.45–16.87)	12.46 (11.34–14.11)	r = 0.45	0.023 *	0.032 *
Antioxidant capacity *	537.50 (456.75–598.50)	550.00 (526.00–645.50)	r = 0.52	0.009 *	0.022 *
Hair cortisol concentration #	10.91 ± 6.17	12.18 ± 6.18	d = 0.26	0.102	0.105
Leukocyte telomere length (T/S ratio) #	1.49 ± 0.39	2.03 ± 0.54	d = 1.53	<0.001 *	0.008 *

* Data are presented as median (interquartile range, IQR). Comparisons were performed using the Wilcoxon signed-rank test. Effect size is reported as r. # Data are presented as mean ± standard deviation (SD). Comparisons were performed using paired-samples *t*-tests. Effect size is reported as Cohen’s d.

## Data Availability

The datasets used and/or analyzed during the current study are available from the corresponding author on reasonable request. The data are not publicly available due to ethical and privacy restrictions related to participant confidentiality.

## Abbreviations

The following abbreviations are used in this manuscript:

HPA	Hypothalamic–Pituitary–Adrenal
HCC	Hair Cortisol Concentration
PSAI	Pythagorean Self-Awareness Intervention
DMN	Default Mode Network
DXA	Dual-energy X-ray Absorptiometry
BMI	Body Mass Index
FFM	Fat-Free Mass
IMAT	Intermuscular Adipose Tissue
PSS-14	Perceived Stress Scale
DASS-21	Depression Anxiety Stress Scales
RSES	Rosenberg Self-Esteem Scale
HLPCQ	Healthy Lifestyle and Personal Control Questionnaire
HLC	Health Locus of Control Scale
STAXI	State–Trait Anger Expression Inventory
SSGS	State Shame and Guilt Scale
PSQI	Pittsburgh Sleep Quality Index
BICAMS	Brief International Cognitive Assessment for Multiple Sclerosis
BVMT-R	Brief Visuospatial Memory Test—Revised
CVLT-II	California Verbal Learning Test—II
SDMT	Symbol Digit Modalities Test
TOS	Total Oxidant Status
BMR	Basal Metabolic Rate
NC	Neck Circumference
WC	Waist Circumference
HC	Hip Circumference
ICW	Intracellular Water
ECW	Extracellular Water
MDTOTAL	Mediterranean Diet Total Score
SD	Standard Deviation
IQR	Interquartile Range
